# Indicators for Reproductive Violence: A Systematized Review to Develop a Multilevel Measurement Framework

**DOI:** 10.1111/sifp.70021

**Published:** 2025-06-20

**Authors:** Erin Pearson, Jasmine Uysal, Sabrina Boyce, Alexandra Robinson, Nora Piay‐Fernandez, Deekshita Ramanarayanan, Sarah Barnes, Jay G. Silverman

## Abstract

The term reproductive violence (RV) unifies forms of gender‐based violence that compromise reproductive autonomy. This study developed a multilevel quantitative measurement framework for RV comprised of indicators at the interpersonal, community, institutional, and law and policy levels. We conducted a systematized literature review, extracting and scoring existing indicators based on prior testing in a low‐ and middle‐income country setting, psychometric data, feasibility, and face validity. The literature review identified 84 peer‐reviewed studies, inclusive of 448 indicators that were extracted and scored. Ultimately, 112 indicators were included in the RV measurement framework. Indicators were organized by level of the social‐ecological model and across three categories: (1) pregnancy‐promoting RV, (2) pregnancy‐preventing RV, and (3) legal and social liabilities of pregnancy and parenthood. This study provides the first multilevel measurement framework for RV. Further research is needed to develop indicators for understudied RV constructs and validate the framework. The resulting framework will be used at the subnational, national, and regional levels to understand barriers to reproductive autonomy.

## BACKGROUND

The reproductive justice (RJ) movement has drawn attention to the ways in which intersecting identities such as race, gender, and class limit people's access to reproductive healthcare and their enjoyment of reproductive rights (Ross and Solinger 2017). The RJ movement has also identified the role that a variety of actors play in limiting access, from healthcare providers to the community and to law and policymakers (Ross and Solinger 2017). Limitations on access to reproductive healthcare become a form of gender‐based violence (GBV) when they control, force, or coerce people into pregnancies or births that they do not want or prevent people from having wanted pregnancies by coercing them into using contraception or by denying them access to fertility treatment due to their identities. Much of the previous work at the intersection of GBV and reproductive health has focused on reproductive coercion, defined as behaviors by a partner or family member that interfere with contraceptive use or pregnancy decisions (Miller, Jordan, et al. 2010; Silverman and Raj 2014). The term reproductive violence (RV) is conceptualized as a unifying theme for forms of GBV that have reproductive impacts, from the interpersonal level (such as reproductive coercion) to the law and policy level (such as state‐sponsored violations of reproductive rights). Despite the long‐documented cases of state‐sponsored reproductive coercion globally (Barot 2012), RV has not been recognized as a form of violence in legal frameworks (Chadwick and Jace Mavuso 2021). In 2024, the United Nations Population Fund (UNFPA) developed a white paper on RV (UNFPA Forthcoming), which defines RV as

A form of gender‐based violence against women and girls that compromises reproductive autonomy, agency and self‐determination; that is, individuals’ ability to decide whether, when, how, and under what conditions to become pregnant, give birth and raise children, as defined at the International Conference on Population and Development (ICPD). This includes, but is not limited to, physical, sexual, emotional, psycho‐social, economic, normative and symbolic abuse, force, coercion or exploitation within relationships, communities, institutions and societies.

Despite growing recognition of the impact that forms of RV have on health and well‐being (Miller, Jordan, et al. 2010; Silverman and Raj 2014), measurement of RV is in a nascent stage. Previous measurement work has focused on interpersonal forms of RV, such as reproductive coercion perpetrated by male partners or family members (McCauley et al. 2017; Silverman et al. 2019). More recently, there has been increased attention on the role of healthcare providers, with measurement focused on quality of person‐centered family planning (Dehlendorf et al. 2018) and abortion care (E. E. Pearson, Chakraborty, et al. 2023; Sudhinaraset et al. 2020; Dennis, Blanchard, and Bessenaar 2017). While important, this approach of measuring only positive aspects of care may miss negative experiences that impact patient experiences and outcomes. Studies that have more specifically focused on violence or coercion in health settings find forms of RV perpetrated by providers, ranging from pressure to use or not use certain contraceptive methods (Senderowicz et al. 2021) to coerced or forced sterilizations (Kendall and Albert 2015). Similarly, studies have identified community‐level stigma related to abortion (Shellenberg, Hessini, and Levandowski 2014) and adolescent family planning use (Hall et al. 2018; Makenzius et al. 2019), pregnancy, and parenting (Hall et al. 2018; Rice et al. 2017) that impact reproductive decisions.

The present study identified and classified RV indicators based on the UNFPA definition and drawn from the existing literature to create a measurement framework for forms of RV that impact the ability to decide whether and when to become pregnant and give birth. Forms of RV that impact how and under what conditions to give birth and raise children, such as obstetric violence, were outside the scope of this review and will be explored in a future phase of this work. The RV measurement framework utilized the social‐ecological model, acknowledging that RV is perpetrated by actors in various contexts across multiple social levels.

## METHODS

### Identification of RV Indicators at the Interpersonal, Community, and Institutional Levels

The study team conducted a systematized literature review to identify studies that described indicators related to RV at the interpersonal, community, and institutional levels of the social‐ecological model. A systematized review provides an initial assessment of the literature but is less rigorous than a systematic review (Sataloff et al. 2021). The review consisted of three stages to select relevant studies: (1) keyword search, (2) title and abstract screening, and (3) full text review. Screening and reviewing were managed using Covidence software.

#### Stage 1: Keyword Search

Pubmed, Embase, and CINAHL were searched for peer‐reviewed papers published in English between 2002 and 2022 that included indicators of RV used in any country. The keywords used varied by database but were created to cover all theorized RV themes (Table 1), including terms from each of the following topical clusters: (1) reproductive health (e.g., “sterilization,” “contraception,” “abortion”), (2) violence or coercion (e.g., “mistreatment,” “abuse,” “coercion,” “force,” “stigma”), (3) perpetrator (e.g., “partner,” “in‐law,” “health provider,” “pharmacist”), and (4) measurement (e.g., “survey,” “interview,” “measure”). Table 1 provides an overview of the specific RV themes that were explored and categorized by perpetrator type.

**TABLE 1:** Keyword themes by perpetrator type

Interpersonal perpetrators	Themes
Partners or family members	Reproductive coercion Pregnancy coercion Contraceptive sabotage Forced or coerced sterilization, abortion, or contraception Restricted access to sterilization, abortion, or contraception Stigmatizing attitudes towards sterilization, abortion, or contraception Coerced gender‐biased sex selection

Community perpetrators	Themes
Community members	Stigmatizing attitudes towards sterilization, abortion, or contraception Stigmatizing attitudes towards adolescent pregnancy and parenthood
Protesters	Restricted access to abortion care Targeting of abortion providers and abortion seekers

Institutional perpetrators	Themes
Healthcare providers/health institutions	Forced, coerced, or incentivized sterilization, abortion, or contraception Restricted access to sterilization, abortion, or contraception Conscientious objection/denial of abortion care Quid pro quo for access to abortion or contraceptive care Denial of accurate sexual and reproductive health information Stigmatizing attitudes towards sterilization, abortion, or contraception Restricted access to fertility treatment Attitudes in favor of reporting and prosecuting pregnant drug or alcohol users
Faith leaders/religious institutions	Restricted access to sterilization, abortion, or contraception Stigmatizing attitudes towards sterilization, abortion, or contraception
Educators/schools	Restricted access to accurate reproductive health information (sex education) Restricted access to reproductive health services Restricted access to education based on pregnancy or parenthood Stigmatizing attitudes towards adolescent pregnancy and parenthood
Law enforcement/legal institutions	Forced or coerced sterilization, abortion, or contraception in correctional facilities Restricted access to sterilization, abortion, or contraception in correctional facilities Targeting of abortion providers and abortion seekers Legal responsibilities of pregnant people towards the fetus

#### Stage 2: Title and Abstract Screening

Titles and abstracts were screened in Covidence software by one study team member for all studies identified through the keyword search. Studies were advanced to full text review if they were (1) on an RV topic and (2) included reporting of results from a quantitative or qualitative study. The study team also included studies from an unpublished systematized review on reproductive coercion indicators conducted in 2020.

#### Stage 3: Full‐Text Review

Studies that were advanced in stage 2 underwent full‐text review by one study team member to determine eligibility for inclusion. Studies were considered eligible if (1) they were a quantitative study and (2) the RV indicator(s) were reported. Quantitative studies that did not report the RV indicators used were excluded. Qualitative studies were included in a separate list of studies for later analysis and development of new indicators to fill gaps in the framework.

### Indicator Extraction and Scoring

**FIGURE 1:**
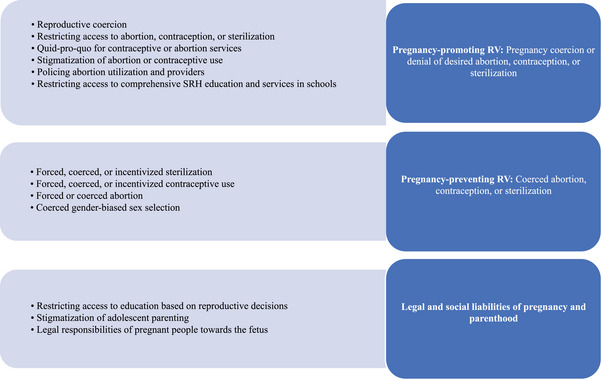
Reproductive violence themes (left) and categorization into forms of reproductive violence (right)

For all studies included in the review, the study team extracted quantitative indicators and recorded the following information in a spreadsheet: (1) the indicator or scale, (2) the indicator source, (3) the perpetrator, (4) the country context in which the indicator was developed and tested, (5) mode of data collection used, (6) population in which it was measured, (7) any prevalence data, and (8) any validity or reliability testing information. Extracted indicators were then categorized by theme (see Table 1) and level of the social‐ecological framework (interpersonal, community, or institutional). These themes were further categorized into three overarching forms of RV: (1) pregnancy‐promoting RV (pregnancy coercion or denial of desired abortion, contraception, or sterilization), (2) pregnancy‐preventing RV (coerced or forced abortion, contraception, or sterilization), and (3) legal and social liabilities of pregnancy and parenthood (Figure 1). Pregnancy‐promoting and pregnancy‐preventing RV are well‐established in the RC literature (Tarzia and Hegarty 2021; E. E. Pearson, Aqtar, et al. 2023; Humphreys and Sheeran 2024), while legal and social liabilities of pregnancy and parenthood are primarily represented in the law and policy literature and include restrictions on access to education due to pregnancy or parental status, stigmatization of adolescent parenting in schools and communities, and legal responsibilities of pregnant people towards a fetus, which have recently received additional attention in the United States with the introduction of fetal personhood measures (McGovern, Memaj, and Rivera 2025).

Once the relevant quantitative indicators had been identified and categorized, a member of the study team scored each indicator on five criteria:

1. Tested in a low‐ or middle‐income country (LMIC) setting (to ensure broad utility),
2. If a scale: Acceptable internal validity/reliability (e.g., Cronbach's alpha > 0.7),
3. Acceptable external validity (e.g., associated with relevant outcomes),
4. Feasible to collect (e.g., indicators collected via population‐based surveys were deemed more feasible to collect than indicators collected via mystery client surveys), and
5. High face validity (e.g., the indicator well‐represented the concept).

Each criterion was scored as 0 (does not meet criterion), 1 (somewhat meets criterion or might meet criterion if more information were available), or 2 (clearly meets criterion). For each indicator, a mean score was calculated across the five criteria (or across the four applicable criteria if it was not a scale). The study team met in a series of technical consultations to review the mean scores for each indicator and come to a consensus on whether it would be recommended for inclusion in the RV measurement framework. This process helped ensure that indicators would be included for all identified RV constructs, where possible. The study's principal investigator was designated as the decision‐maker in the case of disagreements, but group consensus was reached for all indicators.

Finally, among indicators included in the RV measurement framework, the study team made an overall assessment of each indicator's validity, considering the settings in which it had been validated and existing validity and reliability findings. Three categories were used: (1) green signified strong validity, which we defined as adequate to strong validation in two or more LMIC contexts; (2) yellow signified moderate validity, which we defined as adequate to strong validation in at least one LMIC context; and (3) red signified that no validity data existed. Indicators in the red category were included if they represented an important construct that was otherwise missing.

### Identification of RV Indicators at the Law and Policy Levels

A subset of the study team with policy expertise conducted a literature review to identify existing law and policy indicators. Google Scholar was used to search the peer‐reviewed literature for policy frameworks related to the RV definition, including indicators related to reproductive decision‐making power, discrimination based on gender, age, sexuality, marital status, race, ethnicity, and/or incarceration status, and access to sexual and reproductive health (SRH) services and education. This more simplified approach was selected due to the dearth of law and policy studies with quantitative indicators.

## RESULTS

**FIGURE 2:**
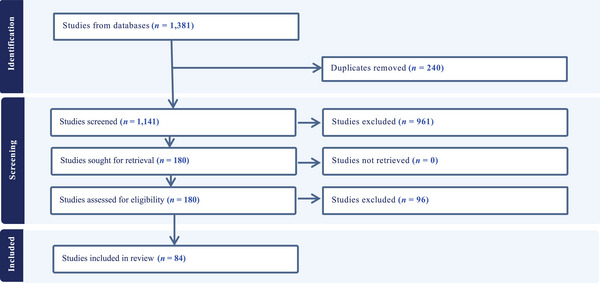
PRISMA flow diagram

The systematized literature review to identify indicators at the interpersonal, community, and institutional levels resulted in the identification of 1381 peer‐reviewed publications, 1141 publications underwent title and abstract screening, 180 underwent full text review, and 84 were ultimately included in the review (Figure 2). A total of 96 studies were excluded at the full‐text review stage because they did not specify the indicators used.

The study team extracted 448 indicators from the 84 peer‐reviewed publications included in the review, including 108 indicators at the interpersonal level, 130 indicators at the community level, and 210 indicators at the institutional level (Table 2). The policy research team also extracted 13 law and policy indicators from 12 peer‐reviewed publications.

**TABLE 2:** Number of indicators identified in the literature review by form of reproductive violence (RV) and level of the social‐ecological model

	Level of the social‐ecological model			
Form of RV	Interpersonal	Community	Institutional	Law and policy
Pregnancy‐promoting RV	88	111	193	10
Pregnancy‐preventing RV	15	0	8	3
Legal and social liabilities of pregnancy and parenthood	5	19	9	0

### Pregnancy‐Promoting RV Indicators

Over 87 percent of the RV indicators identified (402 indicators) were for pregnancy‐promoting RV (Table 2), reflecting the relatively large literature on abortion stigma (112 indicators), denial of abortion care and conscientious objection (103 indicators), and reproductive coercion (59 indicators). For some of the RV themes, only a few indicators were identified, including targeting of abortion providers (eight indicators), restricting access to comprehensive SRH education and services in schools (three indicators), and quid pro quo for abortion services such as requiring additional payment over the cost of the abortion service (one indicator).

### Pregnancy‐Preventing RV Indicators

Only 5.6 percent of identified indicators (26 indicators) were for pregnancy‐preventing RV. Half of these indicators were for coerced gender‐biased sex selection (13 indicators), which originated from a single study (Sattar et al. 2020). The review also identified indicators of provider‐perpetrated contraceptive coercion (six indicators), forced or coerced sterilization (two indicators), and partner‐ or family member‐perpetrated contraceptive and abortion coercion (two indicators).

### Legal and Social Liabilities of Pregnancy and Parenthood Indicators

A similarly small proportion of identified indicators (7.2 percent; 33 indicators) fell under legal and social liabilities of pregnancy and parenthood. The majority of indicators were related to stigma against adolescent parenting (25 indicators), and the remaining eight indicators were related to the use of the criminal justice system to ensure the health of fetuses.

### RV Measurement Framework

After review and scoring, 112 indicators were recommended for inclusion in the RV measurement framework. This included 95 indicators identified in the review and 17 investigator‐developed indicators for RV themes where no existing indicators were identified in the review (15 law and policy indicators, one community‐level indicator for social norms coercing abortion, contraceptives, or sterilization, and one institutional indicator on denial of access to contraceptives). Table 3 shows the distribution of the 112 recommended RV indicators by form of RV and level of the social‐ecological model.

**TABLE 3:** Number of recommended indicators by the form of reproductive violence (RV) and level of the social‐ecological model

	Level of the social‐ecological model			
Form of RV	Interpersonal	Community	Institutional	Law and Policy
Pregnancy‐promoting RV	13	6	46	20
Pregnancy‐preventing RV	3	1	8	5
Legal and social liabilities of pregnancy and parenthood	3	2	2	3

For each of the 112 recommended indicators, the RV measurement framework provides a recommended mode of data collection (e.g., household survey, health facility‐based survey, law/policy review) and a recommended population in which to collect the indicator (e.g., women of reproductive age, women seeking reproductive health services, abortion providers). Finally, the framework includes an overall assessment of each indicator's validity. Among the 112 indicators, only four were categorized as green (strong validity), 11 were categorized as yellow (moderate validity), and 97 were categorized as red (no validity information available). All 56 indicators at the institutional level of the social‐ecological model were categorized as red; despite many indicators in existence (e.g., indicators of conscientious objection), none had published validity data from LMIC contexts. The full measurement framework can be found in Online Appendix A1.

## DISCUSSION

This study identified over 400 RV indicators reported in the peer‐reviewed literature, with most measuring pregnancy‐promoting RV. While some indicators have been widely used and validated in a variety of settings, such as the reproductive coercion scale (McCauley et al. 2017; Silverman et al. 2019, 2020) and the stigmatizing attitudes and beliefs scale (Shellenberg, Hessini, and Levandowski 2014; Holcombe et al. 2018), there are many forms of RV for which indicators lack validity data or do not exist at all. This study developed the first multilevel measurement framework for RV, considering the range of experiences at the interpersonal, community, institutional, and law and policy levels when deciding whether and when to get pregnant or give birth. The measurement framework includes indicators of pregnancy‐promoting RV (pregnancy coercion and denial of reproductive healthcare), pregnancy‐preventing RV (denial of wanted pregnancies and births through forced or coerced reproductive healthcare), and legal and social liabilities of pregnancy and parenthood.

The systematized literature review found that the vast majority of studies have focused on pregnancy‐promoting RV, with less attention paid to pregnancy‐preventing RV and legal and social liabilities of pregnancy and parenthood. We speculate that this imbalance results from the family planning field's focus on pregnancy prevention rather than the broader conceptualization of family planning, which would support people in meeting their reproductive goals, whatever those may be. For example, much of the research on reproductive coercion has focused on pregnancy‐promoting forms of RC such as pregnancy coercion and contraceptive sabotage (McCauley et al. 2017; Silverman et al. 2019, 2020; Miller, Jordan, et al. 2010; Miller et al. 2014; Miller, Decker, et al. 2010), but recently, more studies have expanded the definition of reproductive coercion to include pregnancy‐preventing behaviors such as forced or coerced contraceptive use and abortion (E. E. Pearson, Aqtar, et al. 2023; Tarzia and Hegarty 2021; Moore, Frohwirth, and Miller 2010; E. Pearson et al. 2024). The proposed RV measurement framework includes investigator‐developed indicators for some aspects of these forms of RV at the policy level, such as restricted access to fertility treatment based on age, marital status, gender identity, or sexuality, but further research is needed to develop indicators at the interpersonal, community, and institutional levels to measure infertility‐related violence at these lower levels of the social‐ecological model. Overall, additional research is needed to develop a more robust set of indicators on pregnancy‐preventing RV and legal and social liabilities of pregnancy and parenthood.

The review also found that most previous measurement work has focused on male partners, in‐laws, community members, and healthcare providers as perpetrators. Few indicators were identified for RV perpetrated by faith leaders/religious institutions, educators/schools, and law enforcement/legal institutions, and even fewer indicators were identified to measure RV at the law and policy level. Qualitative studies and expert commentaries have identified these understudied forms of RV (Bent‐Goodley and Fowler 2006; Peterson and Bonell 2018; de Londras et al. 2022; Ethics Committee of the American Society for Reproductive Medicine– 2021), but quantitative indicators are needed to measure RV perpetrated by these actors. For example, the systematized review did not identify quantitative indicators for barriers to reproductive healthcare for gender‐diverse populations, but this is an emerging area of research (Ethics Committee of the American Society for Reproductive Medicine 2021) for which quantitative indicators should be developed and tested for inclusion in the RV measurement framework. This review also found that the majority of indicators focused on acts of violence; while qualitative research has documented survivor experiences (E. E. Pearson, Aqtar, et al. 2023; Moore, Frohwirth, and Miller 2010; Chiweshe, Fetters, and Coast 2021), quantitative indicators of survivor perspectives were lacking. In addition, there has recently been a concerted effort to document RV in humanitarian settings through UN investigative mechanisms (UN Women 2024), and the measurement framework should be expanded to include indicators specific to humanitarian settings, such as pregnancy resulting from conflict‐related sexual violence. Once the RV measurement framework has been completed, it should be validated in both development and humanitarian settings to ensure broad applicability.

The RV measurement framework recommended in this study includes 112 indicators that cover all three forms of RV and all levels of the social‐ecological model. The measurement framework includes fewer indicators in the areas that are well‐studied and more indicators in areas where further research and testing are needed. For example, few indicators of reproductive coercion are proposed as part of the measurement framework because the framework focuses on the widely used indicators that have been validated in a variety of settings (McCauley et al. 2017; Silverman et al. 2019, 2020). Similarly, despite the large number of community‐level RV indicators identified through the review, only the most widely used, validated indicators (e.g., indicators for community‐level abortion stigma; Shellenberg, Hessini, and Levandowski 2014; Holcombe et al. 2018) were included in the measurement framework. This is in contrast to the institutional level, where many indicators, especially for healthcare providers, were included in the measurement framework because few had been validated. For example, though many indicators of conscientious objection to provide abortion services were identified (Harris et al. 2016; Bennett et al. 2020; Puri et al. 2018; Fekadu et al. 2022; Awoonor‐Williams et al. 2018; Jim et al. 2023), the review did not identify any validation studies, and more testing is needed to recommend conscientious objection indicators for the final RV measurement framework.

Important limitations include that this was a systematized review, which has inherent limitations compared to a systematic review, and relevant indicators may have been excluded. In this study, relatively narrow search terms were used to make the indicator extraction process manageable, and only one member of the study team completed title and abstract screening and full text review for each identified publication. In addition, gray literature was not searched, and for understudied forms of RV, such as denial of comprehensive sexuality education, restricting to peer‐reviewed literature likely excluded some existing indicators. The literature review for the law and policy indicators was not systematized. Few existing law and policy indicators were identified through the literature review, and the measurement framework includes investigator‐developed indicators that have not been used previously. Generally, we found that the measurement on RV issues, particularly at the community level and for forms of RV related to legal and social liabilities of pregnancy and parenthood, is underdeveloped in the peer‐reviewed literature. It is likely that there are additional forms of RV, particularly in these categories, which have not been adequately explored in the identified literature or considered by the study team and are thus not included in this framework. We encourage future research to focus on these areas and continue to identify examples of RV and develop validated measures, building on the measurement framework presented here, to fill these gaps. Finally, we acknowledge that quantitative measures of RV, while critically important for capturing the prevalence of this public health problem, are limited in their ability to capture the nuances and complexities of RV, particularly regarding survivors’ lived experiences. Thus, we recommend that RV research pairs quantitative measurement of RV with qualitative and/or ethnographic research and frameworks to ground data in survivors’ lived experiences.

## CONCLUSIONS

This study developed the first multilevel measurement framework for RV. The measurement framework is organized by form of RV and level of the social ecological model, which enables researchers and program implementers to identify the indicators most relevant to their work. Further research is needed to expand the RV measurement framework to include indicators for how and under what conditions to give birth and parent (such as obstetric violence), for understudied perpetrators of RV (such as religious, educational, and legal institutions), for indicators of survivor perspectives on RV, for indicators specific to RV in humanitarian settings (such as pregnancy resulting from conflict‐related sexual violence), and for understudied constructs, including the full range of pregnancy‐preventing RV and other aspects of legal and social liabilities associated with pregnancy and parenthood. Once a more complete RV measurement framework has been drafted, validation studies should be conducted in both development and humanitarian settings. This study lays the foundation for a validated RV measurement framework that can be used at subnational, national, and regional levels to understand barriers to reproductive autonomy and ultimately to improve access to care and ensure justice for survivors.

## Supporting information



Supplement Information
